# Supplementary material for: Association between oral microbiome and five types of respiratory infections: a two-sample Mendelian randomization study in east Asian population
Source: Front Microbiol. 2024 Apr 10;15:1392473. doi: 10.3389/fmicb.2024.1392473 (PMC11039966; doi:10.3389/fmicb.2024.1392473)
Supplement: Supplementary file 1 [file Data_Sheet_1.zip › Supplementary Materials/MR plots of saliva/Chronic sinusitis/p__Patescibacteria/forest.pdf]

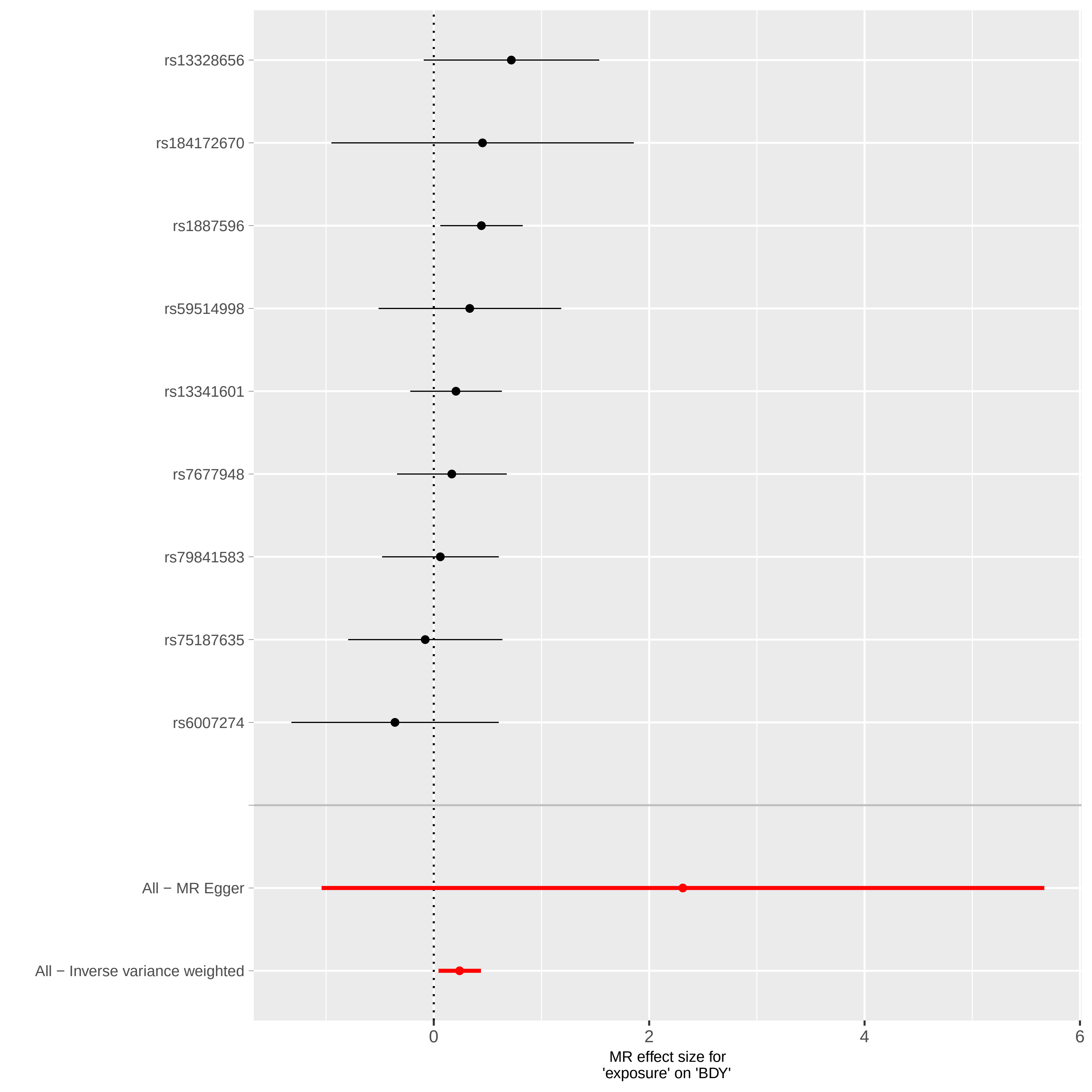
rs13328656
rs184172670
rs1887596
rs59514998
rs13341601
rs7677948
rs79841583
rs75187635
rs6007274
All − MR Egger
All − Inverse variance weighted
0
2
4
6
MR effect size for
'exposure' on 'BDY'
